# Human Papillomavirus and Infertility

**DOI:** 10.3390/medicina55070377

**Published:** 2019-07-15

**Authors:** Vilma Jeršovienė, Živilė Gudlevičienė, Jolita Rimienė, Dalius Butkauskas

**Affiliations:** 1 Santaros Fertility Center, Vilnius University Hospital Santaros Klinikos, Santariskiu Str. 2, 08661 Vilnius, Lithuania; 2 Medicina Practica Laboratory, P. Baublio Str. 4, 08406 Vilnius, Lithuania; 3 National Cancer Institute, Santariskiu Str. 1, 08660 Vilnius, Lithuania; 4 Nature Research Centre, Akademijos Str. 2, 08412 Vilnius, Lithuania

**Keywords:** HPV, in vitro fertilization, semen analysis, infertility, astenospermia

## Abstract

*Background and objectives.* Human papillomavirus (HPV) is the most commonly sexually transmitted infection. Recent evidence suggests that an HPV infection may affect fertility. The aim of the study was to determine the prevalence of HPV infections among couples undergoing in vitro fertilization (IVF) and to identify their awareness of HPV. *Material and Methods.* A total of 200 samples were collected from couples who received IVF treatment during 2017–2018 in Vilnius University Hospital Santaros Klinikos (VUH SK) Santaros Fertility Centre (SFC). For HPV detection, cervical swabs from women and sperm samples from men were taken and a real time polymerase chain reaction (RT-PCR) was used for the identification of 14 high-risk HPV types. Sperm parameters were evaluated according to World Health Organization (WHO) recommendations for 2010. Research subjects answered an anonymous questionnaire to ascertain their knowledge of HPV. *Results.* After testing of HPV in couples undergoing IVF, it was found that 33 out of 100 couples (33%) were HPV positive. Of these, 19% of women (19/100) and 20% of men (20/100) tested positive. Using Fisher’s exact test, a statistically significant difference was found between HPV infections and abnormal sperm quality parameters (*p* = 0.023). *Conclusions.* HPV may have an impact in spermatogenesis, because an HPV infection was more frequently detected in men with abnormal sperm parameters. High-risk HPV 52 was the most common genotype among couples undergoing IVF treatment.

## 1. Introduction

Human papillomavirus (HPV) is a sexually transmitted infection common among men and women of reproductive age worldwide. HPV viruses are associated with epithelial lesions and cancers. HPV infections have been shown to be significantly associated with many adverse effects in reproductive function [1,2].

Infertility is a worldwide problem which affects about 10–30% of couples of reproductive age [3,4]. One of its causes is sexually transmitted diseases. Research over the last few years has been carried out around the world to determine the significance of HPV, which can be an etiological agent that determines miscarriage or infertility. Scientific literature states that HPV infections are three times more common among spontaneous abortion samples than among elective abortions [5]. In one of the clinical studies, women with a cervical HPV infection were reported to have a significantly lower number of pregnancies after assisted reproduction procedures than women with negative HPV results [6]. In his study (2018), K. Zacharis summarized that HPV infections are significantly related to infertility, as an HPV infection has a negative effect on sperm quality (usually astenospermia) and increases levels of antisperm antibodies. In previous studies, it has been observed that if a woman is HPV-positive, there are a higher number of premature ruptures of membranes and spontaneous abortions. If semen is infected with the HPV virus, HPV is thought to affect the acrosomal reaction and lead to reduced acrosome functionality and capacity. The effect of HPV infections on the results of intrauterine insemination (IUI) has also been described. A study of 590 women who had undergone intrauterine insemination showed that those with an HPV infection had six times less pregnancies compared to those who tested negative [7]. Recently, there has been a particular interest in the prevalence of HPV among men. Recent studies have provided evidence of a possible relationship between an HPV sperm infection and idiopathic astenospermia [8,9,10,11]. As a result, there is now more interest in HPV prevalence among couples undergoing in vitro fertilization (IVF). A viral infection is also known to induce blastocyst apoptosis in animal embryos [12]. The scientific literature states that an HPV infection is associated with a higher rate of spontaneous fetal loss or underdevelopment when undergoing IVF [13,14] due to the possible transmission of the virus to oocytes during fertilization, which affects or induces the immune system response. With the increasing number of studies which have analyzed the relationship between HPV infections and male infertility, HPV has been identified as a risk factor for male fertility [15]. Based on this scientific basis, a prospective study was designed to determine HPV infection rates, especially high-risk oncogenic HPV types, among couples undergoing IVF in Vilnius University Hospital Santaros Klinikos (VUH SK).

## 2. Materials and Methods

A sample of 100 couples who received IVF treatment at VUH SK Santaros Fertility Centre (SFC) from October 2017–November 2018 was analyzed. All participants gave informed consent, and the study protocol was approved by the Vilnius Regional Biomedical Research Ethics Committee (No. of Permission: 158200-17-892-402, Date of Approval: 10 January 2017). For women, cervical swabs were collected before the IVF procedure. The remaining sperm after the IVF procedure were used for HPV identification in men. HPV infections in clinical samples were detected by the nucleic acid amplification method in order to detect and differentiate the types of high oncogenic risk HPV 16, 18, 31, 33, 35, 39, 45, 51, 52, 56, 58, 59, 66, and 68 in clinical material (AmpliSens HPV HCR genotype-FRT PCR kit, Federal Budget Institute of Science “Central Research Institute for Epidemiology”, Moscow, Russia). DNA was extracted from 200 samples using the QIAamp DNA Blood Mini Kit (QIAGEN, Hilden, Germany) according to the DNA Purification from Blood or Body Fluids (Spin Protocol) protocol described in the manual QIAamp DNA Mini and Blood Mini Handbook. DNA purification was performed using a Qiagen QIAcube analyzer (QIAGEN Instruments, Hombrechtikon, Switzerland). Before testing, the purified DNA was stored at −20 °C for one week prior to the HPV PCR reaction, which was done using the ROTOR gene 6000^®^ (Corbett Research, Mortlake, Australia) automatic machine. The PCR reaction was prepared according to the instruction manual of the AmpliSens HPV HCR Genotype-FRT PCR Kit; the Amplisens-1 amplification program was used. 

### 2.1. Sperm Analysis

For all men, a sperm analysis was performed to determine its quantitative and qualitative parameters on the basis of World Health Organization (WHO) 2010 recommendations (“WHO Laboratory Manual for the Examination and Processing of Human Semen”, 5th, 2010 [16]). In addition, an anonymous questionnaire survey (*n* = 132) was conducted to investigate male and female knowledge of HPV infections.

### 2.2. Statistical Analysis

A statistical analysis of the findings was performed using IBM SPSS-23.0 software. The results include frequency and percentage frequency tables for the evaluation of the nominal attributes. Fisher’s exact test was used to determine the statistical significance of HPV DNA results and sperm parameters. The differences were considered statistically significant when *p* < 0.05.

## 3. Results

**Figure 1:**
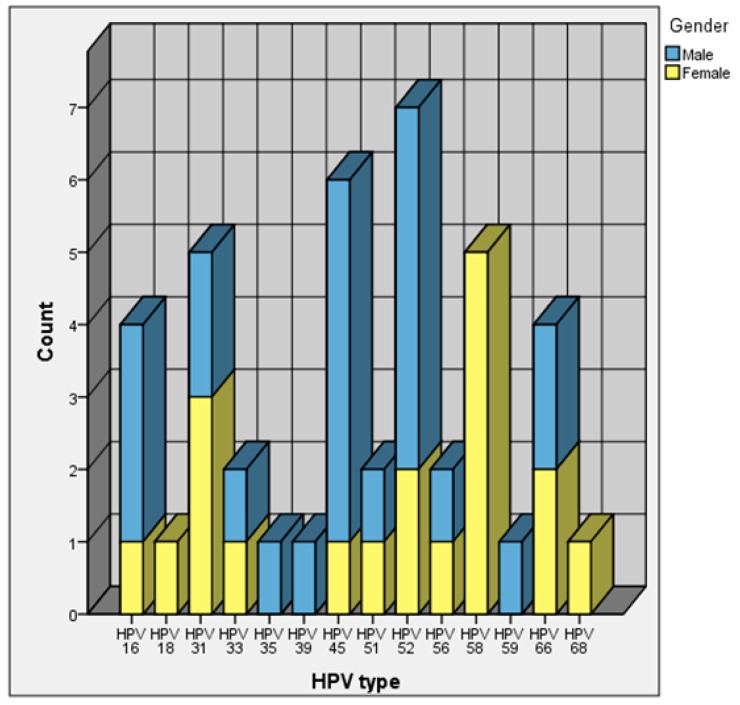
The prevalence of high risk human papillomavirus (HPV) types among males and females (detected during the current study).

One hundred couples undergoing IVF treatment at VUH SK SFC participated in the study (100 samples of cervical swabs and 100 samples of male sperm were collected and investigated in total). The study population age ranged from 26 to 55 years, with the average age of 35.9 years (SD ± 4.95). The average age of women was 35.4 years (SD ± 4.74) and the average of men was 36.4 years (SD ± 5.12). The analysis of the samples for 14 high-risk HPV types using the RT-PCR method revealed the prevalence of HPV infections among 33% of all couples (33/100). According to our study, HPV high-risk types were found in 19% of women (19 out of 100) and 20% of men (20 out of 100). Multiple HPV infections were detected in three semen samples. In seven couples (7%) an HPV infection was found in both partners. The prevalence of HPV types is shown in Figure 1. High-risk HPV 58 was found most often in cervical swabs, accounting for 26% (5/19) of all HPV positive female samples, while high-risk HPV 52 was found in sperm samples, which accounted for 25% (5/20) of all HPV-positive sperm samples. It turned out that HPV 52 was the most common type among couples receiving assisted reproduction treatment, since it was found in seven patients (two women and five men).

For all the patients, semen samples for sperm quality were collected before the IVF procedure, and the residual material was used to identify HPV DNA. Normospermia is a result of semen analysis that shows the normal values of all ejaculate parameters. Some of these parameters are sperm concentration, progressively motile, and morphologically normal spermatozoa. Abnormal sperm has morphological defects (head, neck, midpiece, and tail defects), as well as concentrations or/and progressive motility below the lower reference limits. According to WHO recommendations, the lower thresholds for semen parameters are as follows: semen volume >1.5 mL, sperm concentration >15 million/mL, total sperm number per ejaculate >39 million, sperm progressive motility >32%, sperm morphology using strict criteria >4% normal forms. Normospermia was found in 50% of samples, and another 50% had various sperm quality abnormalities: Low sperm concentration (oligospermia), reduced sperm motility (astenospermia), etc. The prevalence of HPV infections in sperm samples without changes (normospermia) was 10%, and their prevalence was 30% in sperm samples with various changes. The prevalence of HPV infections in sperm samples is presented in Table 1.

Comparing the sperm concentration and motility of HPV positive and HPV negative subjects, it was found that there was a statistically significant difference in sperm quality (*p* = 0.023). Among men with various abnormalities in sperm quality, 75% of HPV-positive samples were found, and 56.2% of HPV-negative samples were found to have no changes in semen. The correlation between HPV infections and sperm parameters is presented in Table 2.

HPV 52 was most commonly found in the case of low sperm concentration (oligospermia) and reduced sperm motility (astenospermia). Semen quality results and detected HPV genotypes are presented in Table 3.

All the men and women participating in the study were invited to fill in an anonymous questionnaire to assess patients’ knowledge of HPV. Two hundred questionnaires were distributed for couples, 132 of which (66%) were completed and returned. Men in the anonymous questionnaire had to answer eight questions related to HPV knowledge and the diseases the virus causes, vaccination, and tests for HPV infections. Sixty out of the one hundred anonymous questionnaires distributed to men were completed and returned. Women were given 12 questions related to HPV, the Cervical Cancer Screening Program, vaccination against the HPV virus, and tests for this infection. Seventy-two out of the one hundred anonymous questionnaires distributed to women were completed and returned.

Summarizing the results of the anonymous survey, 55% of the respondents had higher education. The majority of the respondents (79%) had not been tested for an HPV infection. 47% of the respondents knew about the diseases caused by HPV infections. The majority of the women were aware of and had participated in the Cervical Cancer Screening Program (92%). Of the interviewed women, 8% had been vaccinated against the HPV virus, while none of the men were.

## 4. Discussion

To our knowledge, this is the first study in Lithuania which has investigated the prevalence of HPV infections among couples undergoing IVF treatment and the effect of HPV infections on sperm parameters. The results of this study confirm that HPV infections found in men with infertility problems have a significant impact on their sperm quality. A statistically significant relationship was found between an HPV infection and sperm motility and concentration.

More and more often, scientific literature discusses not only the effect of HPV infections on sperm parameters [10,17] but also on fetal development and the effects of assisted reproduction [6,11,12]. We found that the prevalence of HPV infections among men with infertility problems is 20% in Lithuania. Our results match those in the literature: In cases of unexplained infertility, HPV is found in 10–35.7% of semen [11]. It has been reported that HPV 16, HPV 51, HPV 52, and HPV 45 are found in semen most often [5,17,18]. In our study, HPV 52 was found in 25% of all sperm samples which tested HPV positive. Among women, HPV 58 was most commonly found. Our findings suggest that the prevalence of HPV genotypes may vary depending on the geographical area or country. Additional factors, such as lifestyle or the number of partners, also affect the distribution of HPV genotypes.

Many genetic and external factors determine the fertility of both sexes. These factors affect about 15% of infertile couples in Lithuania as well as in other European countries [19]. Our study did not include an analysis of medical records; therefore, we did not have the opportunity to accurately determine the causes of infertility of those involved in the study. However, the statistically significant results revealed by the study should lead to further research on the prevalence and significance of HPV in sperm quality, while the results of HPV prevalence should be compared more widely with those of fertile couples.

In summary, based on the results of the study, it can be stated that HPV prevalence among couples with infertility problems undergoing IVF treatment is consistent with the data in the scientific literature and confirms the importance of HPV infections for sperm parameters (the frequency of HPV infections correlates with sperm abnormalities such as low sperm count and motility).

## 5. Conclusions

HPV may have an impact on spermatogenesis because abnormal sperm results were more frequently found in HPV-infected men. High-risk HPV 52 was the most common genotype among couples undergoing IVF treatment (18% of all HPV-positive samples), while other high-risk genotypes were less common. HPV 52 was most commonly found in the cases of low sperm concentration (oligospermia) and reduced sperm motility (astenospermia) samples.

## Figures and Tables

**Table 1:** The prevalence of human papillomavirus (HPV) infections in sperm samples.

HPV Results		Sperm Analysis		Total
		Abnormal	Normal	
HPV−	N	35	45	80
	%	70%	90%	80%
HPV+	N	15	5	20
	%	30%	10%	20%
	TOTAL	100%	100%	100%

**Table 2:** Comparison of semen parameters and HPV test results.

HPV Results		Sperm Analysis		Total
		Abnormal	Normal	
HPV−	N	35	45	80
	%	43.8%	56.2%	100%
HPV+	N	15	5	20
	%	75%	25%	100%

**Table 3:** Semen quality results and HPV types.

Semen Quality Result	No of Cases	Detected HPV Types
Normospermia	5	16; 31; 45; 51; 66
Polyspermia	2	45, 59 *; 39
Oligospermia	4	33; 52; 52; 66
Astenospermia	6	16; 35, 45 *; 16, 45 *; 52; 52; 52
Azoospermia	3	31; 45; 56

* Cases of multiple HPV infections.
